# Stem Cell Therapy for Pediatric Traumatic Brain Injury

**DOI:** 10.3389/fneur.2020.601286

**Published:** 2020-12-02

**Authors:** Dana Lengel, Cruz Sevilla, Zoe L. Romm, Jimmy W. Huh, Ramesh Raghupathi

**Affiliations:** ^1^Graduate Program in Neuroscience, Drexel University College of Medicine, Philadelphia, PA, United States; ^2^Department of Neurobiology and Anatomy, Drexel University College of Medicine, Philadelphia, PA, United States; ^3^Department of Anesthesiology and Critical Care, Children's Hospital of Philadelphia, Philadelphia, PA, United States

**Keywords:** pediatric TBI, stem cells, progenitor cells, transplantation, behavior, white matter injury

## Abstract

There has been a growing interest in the potential of stem cell transplantation as therapy for pediatric brain injuries. Studies in pre-clinical models of pediatric brain injury such as Traumatic Brain Injury (TBI) and neonatal hypoxia-ischemia (HI) have contributed to our understanding of the roles of endogenous stem cells in repair processes and functional recovery following brain injury, and the effects of exogenous stem cell transplantation on recovery from brain injury. Although only a handful of studies have evaluated these effects in models of pediatric TBI, many studies have evaluated stem cell transplantation therapy in models of neonatal HI which has a considerable overlap of injury pathology with pediatric TBI. In this review, we have summarized data on the effects of stem cell treatments on histopathological and functional outcomes in models of pediatric brain injury. Importantly, we have outlined evidence supporting the potential for stem cell transplantation to mitigate pathology of pediatric TBI including neuroinflammation and white matter injury, and challenges that will need to be addressed to incorporate these therapies to improve functional outcomes following pediatric TBI.

## Introduction

Traumatic brain injury (TBI) is the leading cause of death and disability among the pediatric population. In the United States, TBI affects half a million children under the age of 14 years old each year (1–4). Advancement in acute neurosurgical interventions and neurocritical care have led to a decrease in mortality rates over the past decade (5). However, there are a lack of targeted therapies to limit the life-long cognitive, psychosocial, and emotional deficits seen in pediatric TBI patients (5–7). Preclinical studies utilizing stem cell transplantation therapies have shown efficacy in mitigating brain injury pathology in various models of adult TBI and more recently, pediatric brain injuries. This review summarizes what has been learned about the effects of endogenously expressed and exogenously administered stem cells on recovery from pediatric brain injury, and remaining obstacles that need to be resolved in order to improve the safety and efficacy of stem cell therapy in survivors of pediatric TBI.

### Behavioral Deficits Following Pediatric TBI

Traumatic brain injury sustained during childhood can result in long-term visuomotor, cognitive, and behavioral symptoms that cause lifelong disability and impaired quality of life for survivors of childhood TBI. Pediatric TBI patients commonly exhibit impairments in visual perception, gross, and fine motor function (8–11), which can persist for several years following injury (9, 11). Pediatric TBI patients also exhibit reduced global intellectual functioning, attention, and processing speed which can lead to deficits in learning and acquiring new information (12–15). Up to fifty- percent of children who sustained a TBI were found to have reduced intellectual ability, memory problems, and lower academic achievement relative to healthy peers (12, 16). Impairments in executive functions such as processing speed and inhibitory control and reduced adaptive functioning can persist up to 7–10 years post-injury (12, 14, 17, 18). Brain-injured children and adolescents are at heightened risk of impairments in socialization and communication (9, 15) and are more likely to exhibit new-onset psychological disorders such as anxiety and mood disorders, post-traumatic stress disorder, and antisocial behavior relative to typically developed school-age children (19–25).

Pediatric TBI has been studied using a variety of preclinical models, including controlled cortical impact (CCI), fluid-percussion injury (FPI), closed-head injury (CHI), weight-drop injury, and rotational injury in rodents of ages ranging from postnatal day 7–28 (26–33). Experimental TBI often results in sensorimotor impairments, typically characterized by decreased duration in a rotarod test (28, 29, 33) and/or decreased locomotor activity in an open field test (29), resembling the visuomotor impairments observed in pediatric TBI patients. Hippocampal-based learning and memory deficits are also common following brain injury in immature animals (28–32, 34). These cognitive deficits are often present at chronic time-points post-injury (31, 32) validating the concept that brain injury in infancy results in the children “growing into the learning and memory deficits.” Although the emotional and psychosocial consequences of pediatric TBI have historically received less attention in animal studies relative to cognitive outcomes, a few recent studies have investigated these outcomes after pediatric brain injury. Increased anxiety-like behavior in the elevated plus maze was reported 3 weeks following contusive head trauma in 7-day-old rats (35), and 4 weeks after rotational TBI in 12-day-old mice (33). Deficits in social behavior were reported in adulthood following TBI in 21-day-old mice (36). Preclinical studies of pediatric TBI have begun to place more emphasis on developing a variety of cognitive and psychological tests to measure deficits in brain-injured animals as they age into adolescence and adulthood.

### Pathologic Alterations Following Pediatric TBI

The major pathologic hallmarks of TBI in infants and children include cerebral edema, extra-axial and intraparenchymal hemorrhage, ventriculomegaly, and diffuse axonal injury (DAI) (37). Reductions in cortical, hippocampal and thalamic tissue volume is often observed in neuroimaging studies following TBI in children and correlated with deficits in cognitive function (38–40). Post-traumatic inflammation is thought to be a significant contributor to histopathology following pediatric brain injury (41). Pediatric TBI patients also exhibit elevated levels of pro-inflammatory cytokines in the cerebrospinal fluid (CSF) in the first 3 days after brain injury, particularly in cases of severe TBI (42, 43). Together, these observations underscore the complicated nature of the cellular and tissue pathology in brain-injured children.

Preclinical TBI studies have demonstrated diffuse damage in white matter tracts (27, 31, 44), brain edema (27), ventriculomegaly (31), subarachnoid hemorrhage (27), inflammation (27, 30, 33), and regional reductions in tissue volume (30, 31), resembling the pathology typically observed in patients. Evidence of axonal injury is commonly observed following brain trauma (27, 31), in addition to reductions in myelination volume in the corpus callosum (45), resulting in functional white matter deficits. Deficits in compound action potential (CAP) of myelinated fibers in the corpus callosum at 24 h persisting to at least 2 weeks following closed head injury in the 17-day-old rat have been reported (46). Moreover, contusive injury in 16–18-day old rats resulted in an impairment in the ability to induce long term potentiation in the contralateral somatosensory cortex by stimulating the corpus callosum 2–3 weeks post-injury (44). Observations from both clinical and preclinical studies suggest that the hippocampus is particularly vulnerable to injury-induced pathological changes. Preclinical studies have demonstrated decreased tissue volume (26) in addition to apoptotic cell death (32), neurodegeneration (34, 47), and reactive astrocytosis (27, 30) within the hippocampal formation. Contusive trauma in 17-day-old rats resulted in decreased dendritic length and branching in the CA1 area of the hippocampus in the injured hemisphere (48). Although less extensively studied in pediatric relative to adult TBI, deficits in hippocampal long-term potentiation (LTP) were reported in both the dentate gyrus and CA1 area of the hippocampus between 1 and 4 weeks after weight-drop injury in juvenile rats (49). Impairment in LTP induction in the dentate gyrus was sustained over a longer period following injury in female rats compared with their male counterparts, whereas impairment in LTP induction in the CA1 area was observed only in male rats (49).

Activation of inflammatory sequelae in both the acute and chronic post-injury phases occur following TBI in immature animals. Concentrations of pro-inflammatory cytokines, including interleukin-1β, interleukin-6, and tumor necrosis factor-alpha (TNF-α) are acutely and rapidly elevated in the injured brain (30) and can remain elevated for several days post-injury (33). Brain regions which are particularly vulnerable to post-traumatic inflammation and microglial activation include the peri-injury region of the cortex, corpus callosum, thalamus, and hippocampus (27, 34), which are thought to contribute to sustained cognitive deficits observed following pediatric TBI. Although activation of neuroinflammation can be detrimental following injury to the developing brain, post-injury inflammatory response in the acute post-traumatic period has been suggested to be beneficial for the clearance of cellular debris (50). Collectively, these data provide evidence of the multiple cellular processes that can be targeted for treatment strategies for pediatric TBI particularly in the chronic post-traumatic period.

## Effects of Pediatric TBI on Endogenous Stem Cell Populations

Preclinical evidence suggests that injury to the immature brain has robust effects on the proliferation of endogenous stem cell populations [reviewed in Niimi and Levison (51)]. Multiple studies have sought to identify and characterize the distinct stem cell populations that are affected following brain injury which may be a critical factor in developing therapeutic strategies to replenish cells that are lost following brain injury and to enhance the inherent proliferative capabilities of stem cells following pediatric TBI. The location, type, and population density of neural stem cells (NSCs) are developmentally regulated and characterizing the responses of NSCs and neural progenitors (NPs) in the immature brain to injury can aid in the understanding of brain injury pathology and the development of therapeutic targets for pediatric TBI (51, 52).

### Neural Stem Cells

The response of neural stem cells (NSCs) to neonatal HI is complex. In the early post-injury phase, increased mitotic activity in the subventricular zone (SVZ) is observed (53–55). However, not all cells within this proliferative zone respond to HI similarly as the oligodendrocyte progenitors undergo apoptotic cell death (56); resulting in extensive cell death within the SVZ during the first 24–48 h (56). Although NSCs and glial-restricted progenitor cells survive the injury, PSA-NCAM+ cells (stem cells) as well as late-stage oligodendrocyte progenitors (OPCs) were vulnerable (57–60).

Contusive TBI to the immature brain also found evidence of robust proliferation in response to injury (52, 61). In the immature rat, contusive trauma resulted in an increase in the number of neurospheres, an increase in frequency of NSCs and an accelerated growth rate (52). More recently, Zhang et al. (61) reported decreased survival rates of adult-born neurons along with an increase in ectopic migration of adult neurons in the hippocampus following neonatal brain trauma in rabbits. Additional efforts must be directed to better characterizing the effects of TBI on regional responses of the NSCs and NPs.

Despite the robust proliferative response of NSCs and NPs to pediatric brain injury, evidence suggests that most of the newly generated cells do not survive past 1–2 months (52), or predominantly become interneurons or astrocytes rather than mature neurons (62). A significant increase in proliferating glial fibrillary acidic protein (GFAP)-positive cells occurs after both neonatal HI and pediatric TBI, indicating increased astrocytic proliferation and astrogliosis (62). A few studies have also reported an increase in the number of newly generated oligodendrocytes that presumably originated within the SVZ following HI (63–65). Despite evidence for increased production of oligodenderocytes, impaired myelin production, and axonal loss in the subcortical white matter persist following pediatric brain injury, suggesting that there may be a deficit in the maturation of these newly generated oligodendrocytes (51). In part, this deficit may be driven by increased astrocyte proliferation and associated production of toxic substances that are known to inhibit oligodendrocyte differentiation and the maturation of oligodendrocyte progenitors such as chondroitin sulfate proteoglycans (66). Thus, finding new mechanisms to enhance the long-term survival of newly generated neurons and oliogodendrocyte will be critical for advancing stem-cell based therapies to treat pediatric TBI.

### Peripheral Stem Cells

The bone marrow niche is home to hematopoietic stem cells (HSCs) which are responsive to pediatric brain injuries and play a role in stem cell proliferation, mobilization, and migration. Several studies have found that neonatal HI mobilizes mesenchymal stromal cells (MSCs) in the peripheral blood which migrate to the location of the injured tissue where they can potentially aid in tissue repair and regeneration (67–69). In part, this mobilization and recruitment may be mediated by the actions of stromal cell-derived factor 1 (SDF1) (70) or stem cell factor (SCF) both of which increase in expression within the hippocampus, corpus callosum, and periventricular areas between 3 and 7 days following neonatal HI (69, 71–73).

## Stem Cell Transplantation In Pediatric TBI

A growing body of literature has accumulated supporting the potential of NSCs (either embryonic or adult) and/or MSCs (from either the bone marrow or umbilical cord) to treat the pathophysiology of TBI [reviewed by Mashkouri et al. (68)]. The self-renewal ability of NSCs and inherent potential to differentiate into neurons and glial cells provides the potential to promote regeneration and neurogenesis in the injured brain (74), while MSCs are advantageous due to their ability to cross the blood brain barrier (BBB), migrate to the site of injury, and secrete anti-inflammatory and trophic factors that protect against cell death (67). These approaches have demonstrated that stem cell therapy is effective in mitigating neuronal cell death and inflammatory cascades following TBI, resulting in improved recovery of cognitive and motor functions (67, 68, 74). Although these strategies have been employed in models of adult TBI, there is a growing body of literature supporting similar beneficial effects of stem cell treatments using models of pediatric brain injury. For example, stem cells derived from umbilical cord blood were found to mitigate neurovascular injury following neonatal brain injury (75). Evaluating the effects of stem cell therapies in these neonatal brain injury models will provide important guidance for directing future research on developing targeted and effective stem cell treatments for pediatric TBI.

### Survival of Cells

Evidence from adult animals suggests that cells grafted in the hostile environment of the injured brain often exhibit poor survival (76, 77). In the interest of improving the translational value of using stem/progenitor cells, systemic routes have been tested for efficacy of cell therapy although intravenously administered stem cells can become localized in peripheral organs (such as the lungs or the spleen) rather than migrating to the brain (78). Limited evidence from pediatric animal studies suggests that NSCs can survive and differentiate into neurons, astrocytes, and oligodendrocytes in the injured brain. Long-term survival and differentiation of implanted NSCs was observed 4–5 weeks after intraventricular (79) or intranasal (80) following HI in neonatal mice. Differentiated NSCs were observed up to 2 months after transplantation into the CA3 area of the hippocampus 2 weeks following global ischemia in adolescent rats (81). Intravenously administered MSCs were only observed in the contralesional hemisphere following HI in 1-week-old rats, while beneficial effects of MSC treatment on recovery of motor function, brain volume, cortical thickness, and neuronal density were observed (82). Intranasal administration of stem cells has become an attractive and minimally invasive method to deliver cells into the injured brain that has shown efficacy in preclinical studies of neonatal HI (80, 83, 84) and will likely be more commonly utilized in future research.

### Histopathology and Inflammation

Although enhancing the long-term survival and integration remains an important goal of stem cell therapy, accumulating evidence has highlighted the importance of trophic support and/or anti-inflammatory effects in facilitating the beneficial actions of stem cells in treating the injured brain. NSCs can secrete neurotrophic factors such as nerve growth factor (NGF) and brain-derived neurotrophic factor (BDNF) (85) which promote repair processes and plasticity. MSCs are also known to exert beneficial trophic factors (86–88) as well as anti-inflammatory and antioxidant effects (57, 89, 90) which can attenuate systemic inflammation, a key aspect of pediatric brain injuries. Based on *in vitro* studies using co-cultures of glial-restricted precursor cells (GRPs) with neonatal brain slices exposed to oxygen-glucose depravation, GRPs decreased tissue injury and cortical cell death without direct cell-cell contact suggesting that these effects were likely attributable to trophic support provided by the cells (91). Following HI in neonatal rats, administration of NSCs between 1 and 3 days after injury reduced infarct volume (79, 80) and decreased the number of apoptotic cells in the hippocampus and cortex (79). The administration of MSCs 24–48 h following brain injury in neonatal rats significantly reduced cortical neuronal pathology (92, 93), and the extent of tissue loss (84). Moreover, MSC-administered groups exhibited an increase in the thickness of the cortex (82) and corpus callosum (82, 94) as well as increased neuronal density within the hippocampus (84) compared with vehicle-treated brain-injured animals. MSC administration has also resulted in robust anti-inflammatory effects in models of neonatal HIE. MSC treatment decreased the number of activated microglia in the hippocampus and cortex (83, 84, 94) and inhibited pro-inflammatory cytokine expression (92, 94, 95) in addition to increasing anti-inflammatory cytokine expression (92) in the injured brain.

### Functional Outcomes

Efficacy of cell therapy needs to be documented via their effects on behavioral outcomes. Compared with vehicle-treated animals, MSC-administered animals exhibited improved motor function in the rotarod and beam walking tests between 2 and 4 weeks following brain injury (82, 84, 92, 94). Intranasal administration of MSCs 3 days after HI in neonatal mice also reversed cognitive deficits in the novel object recognition test 5 weeks following injury (83), and MSCs administered into the injured hemisphere 24 h following HI in neonatal rats improved spatial learning at 4 weeks following injury (93). Treatment with NSCs similarly improved spatial learning and memory in addition to motor function 2–6 months following brain injury (79–81). MSC administration reversed risk-taking behavior observed in vehicle-treated injured animals in the elevated plus maze 5 weeks following HI in 9-day-old mice (83). One study found that transplantation of hypoxia-preconditioned NPs increased social interaction, sociability, and social recognition following contusive TBI, which was associated with increased expression of oxytocin and oxytocin receptor compared with vehicle-treated animals (96) suggesting that stem cell treatments may also improve psychosocial outcomes after pediatric brain injury.

### White Matter Injury

Bone-marrow derived MSCs (BM-MSCs) administered following adult TBI or neonatal HI demonstrate beneficial effects on white matter repair and regeneration (95, 97–99). Intravenously administered BM-MSCs following contusive TBI in adult rats promoted structural recovery of white matter indicated by increased fractional anisotropy and axonal water fraction analyzed with diffusional kurtosis imaging (98). MSCs may also be beneficial in mitigating white matter injury following HI presumably by playing a supportive role in oligodendrocyte development (100). Administration of MSCs following neonatal HI increased myelination and expression of myelin basic protein (MBP) (95, 97, 99), reduced microglia and astrocyte activity (99), and decreased pro-inflammatory cytokine expression (95). Thus, stem cell treatments utilizing MSCs would likely be similarly beneficial for mitigating neuroinflammation and diffuse axonal injury following pediatric TBI.

### Neurovascular Injury

Stem cells derived from umbilical cord blood demonstrate great therapeutic potential due to their ability to mitigate damage to the neurovascular unit following brain injury (75, 101). Treatment with umbilical cord-derived endothelial progenitor cells (EPCs) following HI in 7-day-old rats decreased blood-brain barrier damage, brain tissue loss, and deficits in motor function (101). Injured animals infused with umbilical-vein derived EPCs exhibited less apoptosis in the cortex 24 h following injury, which was associated with increased expression of stromal cell-derived factor 1 (102). Transplantation of umbilical cord blood CD34+ cells 1 week following HI in 7-day-old rats improved motor function and decreased the expression of GFAP and apoptotic genes in the injured brain (103). Although EPCs are potentially neuroprotective by facilitating angiogenesis and neovascularization, they may increase BBB permeability—presumably via increasing the local concentration of vascular endothelial growth factor, further exposing the brain to systemic toxins and inflammatory mediators (75, 104).

### Combinatorial Treatments Utilizing Stem Cells

The effectiveness of stem cell treatment combined with other pharmacotherapies in preclinical models of TBI is currently being established. These combination therapies may exhibit synergistic effects and result in greater neuroprotection relative to stem cell or pharmacological monotherapy (79, 105). Moreover, some combination therapies may enhance the viability and survival of implanted stem cells in the injured brain (76, 79).

#### Anti-inflammatory Agents

Anti-inflammatory therapies such as minocycline show promise in adult TBI studies (68), although excessive doses have resulted in worse outcomes, highlighting the importance of the acute inflammatory response to brain injury. Nonetheless, combined treatment with BM-MSCs and minocycline 24 h following cerebral ischemic injury in adult rats resulted in the most histological and functional improvement compared to treatment with MSCs or minocycline alone, including reduction in cell degeneration and increased neuronal density within the hippocampus (106). However, we have previously demonstrated that acute minocycline treatment following closed-head-injury in the neonate rat may exacerbate neurodegeneration and cognitive deficits (47, 107), suggesting that age-at-injury is an important consideration for developing combination stem cell treatments in pediatric TBI.

#### Granulocyte-Colony Stimulating Factor (G-CSF)

Another promising candidate for treatment of TBI is granulocyte-colony stimulating factor (G-CSF), which has an inherent capability to reduce brain edema and promote functional recovery following TBI. G-CSF also promotes neuroprotection of implanted stem cells (108) and recruitment of endogenous stem cells within the bone marrow into the blood stream where they can migrate to the injured brain (68). Combined treatment with umbilical cord blood cells and G-CSF exhibited synergistic effects on inflammation, neurogenesis, and hippocampal cell loss, in addition to more robust and long-term functional recovery following CCI in adult rats (105). Following HI in neonatal rats, treatment with G-CSF decreased apoptotic cell death (109), enhanced neurogenesis and improved cognitive function (110). Although the effects of G-CSF in combination with stem cell treatment have not been evaluated in pediatric studies, combined treatment with G-CSF and stem cell factor following HIE in 7-day-old rats exhibited similar synergistic effects, resulting in decreased brain tissue atrophy and improved motor function (111). Thus, combined treatment with G-CSF and stem cells would likely have beneficial effects in pediatric HIE and TBI and should be explored further.

#### Mild Hypothermia

Mild hypothermia (HT) is currently the only therapy approved for the treatment of neonatal HI. Mild HT usually involves cooling the body to 32–35 degrees Celsius for 12–72 h within the first 6 h after HI (112). In clinical settings, mild HT has been shown to decrease morbidity and mortality in infants exposed to HI, although many infants still suffer from brain damage and disability even with HT treatment (112). In preclinical studies, HT reduced apoptotic cell death, infarct volume, and improved functional outcomes following HI (79, 83). Preclinical studies have begun to evaluate the effects of combined treatment with hypothermia and stem cells in neonatal HI. Mild HT combined with NSC transplantation 24 h following HI in 1-week-old mice resulted in a greater reduction in apoptotic cells in the hippocampus and cortex, smaller infarct volume, and improved neurological function compared to either treatment alone (79). Moreover, HT-NSC treated mice exhibited greater survival and differentiation of NSCs into mature neurons (79), suggesting that hypothermia treatment may increase the survival and long-term integration of NSCs in the injured brain. Compared with rats treated with HT or MSCs alone, HT combined with MSC treatment following HI in 7-day-old rats exhibited the greatest improvement in cell death, gliosis, inflammation, and motor function (113). However, a more recent study reported worse outcomes when HT (delivered at 4 h after injury) was combined with delayed treatment with MSCs, delivered intranasally 3 days following HI injury in neonatal mice (83). While both HT and MSC treatment alone prevented reductions in myelin basic protein (MBP) expression and neuronal density within the hippocampus and striatum, these effects were attenuated when the treatments were combined (83). Injured mice exhibited cognitive deficits in the novel object recognition test that were reversed by MSC treatment alone, but not by HT or combined treatment (83). Thus, further evaluation of the effects of stem cell treatment and HT will be required in order to develop an effective combination therapy to treat pediatric brain injury.

#### Hypoxic Preconditioning of Implanted Stem Cells

Interestingly, there is recent evidence to suggest that hypoxic preconditioning could increase the therapeutic potential of stem cells (96, 114, 115) and may enhance the regenerative capacity of endogenous NSCs in the SVZ (116). Hypoxic preconditioning followed 24 h later by HI in 1-day-old piglets resulted in a significant increase in neurogenesis in the SVZ compared with normoxic controls, which was observed up to 1 week following preconditioning (116). In a murine model of ischemia, hypoxic preconditioning increased the survival and proliferation of transplanted MSCs which was mediated by suppression of apoptotic signaling and facilitation of the secretion of angiogenic factors (114). Thus, hypoxic preconditioning may be one strategy to enhance the survival and proliferative capabilities of implanted cells within the injured host environment.

#### Modification of Stem Cells to Express Neurotrophic Factors

Modifying stem cells to overexpress neurotrophic factors such as BDNF could maximize the inherent neurotrophic benefits of stem cell treatments in pediatric brain injuries (117, 118). BDNF promotes neurogenesis and synaptic plasticity, and reductions in BDNF in the injured brain are observed following both TBI and neonatal HIE (84). Recent evidence indicates that BDNF may enhance myelin regeneration after injury through the TrkB receptor (119, 120). In a rodent model of CNS demyelination, the TrkB agonist LM22A-4 increased myelin thickness and the density of oligodendrocytes 1 week following injury (120). The effects of treatment with stem cells overexpressing BDNF has been evaluated following neonatal stroke. Intranasal delivery of MSCs alone or MSCs overexpressing BDNF (MSC-BDNF) following neonatal stroke were equally effective in reducing infarct size and white matter injury, and both treatments induced cell proliferation in the injured hemisphere (117). However, injured rats treated with MSC-BDNF exhibited additional improvements in motor function at 2 weeks following injury compared with rats treated with MSCs, although these differences were no longer observed at 4 weeks following injury (117). Similarly, intranasal administration of NSCs or NSCs overexpressing basic fibroblast growth factor (NSC-bFGF) following neonatal HI were similarly effective in reducing infarct volume and improving motor function 3–5 weeks following injury (80). Notably, NSC-bFGF had the added benefit of increasing the number of NSCs that differentiated into neurons within the hippocampus and cortex at 35 days after injury relative to the NSC only treatment group (80), suggesting that neurotrophic factors may help promote that survival and integration of implanted NSCs.

#### Stem Cell-Derived Exosomes

A novel therapeutic approach to treat brain injuries has been the use of exosomes derived from stem cells. Exosomes are extracellular vesicles that are produced from the plasma membrane and released into the extracellular space, where they can participate in intracellular signaling through several mechanisms (121). Secreted exosomes can be taken up by other cells via membrane fusion, endocytosis, or ligand-receptor interactions, where their contents can then be released within the target cells (121). In a swine model of TBI, MSC-derived exosomes given during the first 2 weeks post-injury decreased the neurological severity score, and brain-injured animals given exosomes exhibited a significantly shorter time to complete neurologic recovery (122). Moreover, MSC-derived exosomes attenuated post-injury inflammation and improved motor function following CCI (123, 124). The effects of MSC-derived exosomes have also been evaluated in neonatal HI. A combination of HI and neuroinflammation was induced in 2–3-day old rat pups through lipopolysaccharide injection, followed 2 h later by HI. Exosomes derived from umbilical cord-derived MSCs were delivered intranasally in between the inflammatory and HI insults. They observed that the exosome-treated injured group exhibited less expression of inflammatory cytokines and neuronal cell death 24 h following injury, and improved spatial learning in the Morris water maze 4 weeks following injury (125, 126). In a different study, MSC-derived exosomes were delivered via *in utero* intravenous administration following global HI in ovine fetuses. In contrast to the previous study, they did not observe an effect of the exosome treatment on post-injury inflammation (127), although exosome treatment reduced seizure activity and hypomyelination in the injured brain 1-week post H-I (127).

## Summary

TBI remains a leading cause of death and disability in infants and children which lacks treatment options to mitigate the debilitating life-long symptoms experienced by survivors. Stem-cell based therapies can take advantage of the robust proliferative capacity and plasticity in the immature brain, and thus have potential to become an effective treatment for pediatric TBI. Although the beneficial effects of exogenous stem cell transplantation have been established in adult TBI and stroke models, additional research is necessary to determine whether these benefits of stem cell therapy translate to pediatric TBI. Treatments for pediatric TBI require unique considerations that may differ from adult TBI due to differences in the spatial and temporal dynamic of the brain injury. Thus, certain challenges and limitations still need to be overcome in order to translate stem cell treatment methods from adult to pediatric TBI. For example, exogenous stem cells are often transplanted directly into the cavity resulting from TBI in the adult brain. However, this approach is less ideal in the pediatric brain where there is less likely to be an overt cavity or lesion. Therefore, systemic administration of cells through intravenous or intranasal methods has been more commonly utilized in models of pediatric brain injury. Intravenous administration is also desirable because it can be easily translated to the clinical setting as a non-invasive method to deliver stem cells.

Developmental differences between the immature and adult brain also complicate the translation of stem cell treatments to pediatric TBI models. For instance, although the neuroinflammatory response is a key aspect of TBI, the localization and function of microglia during early development differs from the adult brain. Previous evidence suggests that the white matter of the human fetus/infant is densely populated with activated (CD-68+) microglia (128). During very early postnatal development, microglia play important roles in white matter development as well as neuronal differentiation and survival, as they are a crucial source of neurotrophins such as NGF and BDNF (129). Thus, while anti-microglial treatments such as minocycline in combination with stem cell therapy have shown beneficial effects in adult TBI (68), depletion of microglia during postnatal development increases the number of apoptotic in the cerebral cortex (129) and exacerbates neurodegeneration after neonate TBI (50).

The immature brain is also particularly vulnerable to enhanced levels of pro-inflammatory cytokines and free radicals produced by microglia after brain injury due to the reduced antioxidant capacity of the developing brain as well as the vulnerability of OPCs within the white matter tracts (129, 130). Recent studies utilizing administration of exogenous stem cells have demonstrated numerous beneficial effects on post-injury histological, cognitive, and behavioral outcomes in models of pediatric brain injury. In particular, BM-MSCs and umbilical-cord derived blood cells (UCBC) have shown promising results in preclinical models of neonatal HI through their potent anti-inflammatory effects and trophic support. Thus, it is likely that pediatric TBI will be amenable to these stem cell therapies due to overlapping pathologies with neonatal HI. However, further investigation of these treatments will be necessary to determine their effectiveness as well as potential risks in models of pediatric TBI.

The effects of stem cell treatments in adolescent models of TBI have not been investigated, although transplantation of NSCs improved functional outcomes following global ischemia in adolescent rats (81). TBI during adolescence has deleterious effects on developmental processes such as synaptogenesis and synaptic pruning that are critical during this period (131–133). For example, TBI in juvenile rats led to increased neuronal complexity and spine density of pyramidal neurons the prefrontal cortex, suggestive of disruption of normal pruning processes (133). These effects may be influenced in part by the aberrant effects of TBI on microglial activation and function (132). Pediatric TBI increases the number of activated microglia in the injured brain (107, 132). Moreover, microglia can become chronically activated after TBI, residing in a “primed” state that can further exacerbate inflammatory responses and negatively impact functional outcomes (132). These shifts in microglial morphology toward an activated state can also hinder their normal homeostatic role in CNS development, including microglial-dependent synaptic pruning observed in the thalamus, cerebellum, and hippocampus (132). Aberrant synaptic pruning has been strongly linked to neurological dysfunction and is associated with numerous developmental psychiatric disorders including Schizophrenia and Autism Spectrum Disorder (131, 132). Thus, stem cell treatment may have potential in mitigating neurological and behavioral dysfunction in adolescent TBI due to well-documented anti-inflammatory effects and inhibition of microglial activation following brain injury (83, 84, 94) which should be explored further.

A promising therapeutic approach that would be well-suited for pediatric TBI patients is to target the survival of endogenous stem cells, as this would take advantage the robust proliferative capacity in the immature brain in response to TBI (52). This approach is also desirable because it can avoid certain limitations associated with transplantation of exogenous cells. One of the main challenges pertaining to the responses of endogenous stem cells is the poor long-term survival and maturation of these cells after brain injury, particularly of NSCs and OPCs (62). Thus, optimizing strategies to increase the retention and integration of newly generated neurons and oligodendrocytes would be greatly advantageous for treating pediatric TBI. TBI in immature animals and children affects the hypothalamic pituitary adrenal-axis leading to chronically elevated levels of stress hormones (35, 134). Elevated brain concentrations of corticosterone increase cell death in the hippocampus (135) and thus may further contribute to the hostile environment impeding the survival of implanted cells following TBI. Various types of drugs, growth factors, and other signaling molecules can improve neurogenesis and the recovery of cognitive function after TBI (70, 72, 73). Although the majority of these agents have been limited to adult studies, a number of signaling molecules which regulate stem cell mobilization have been evaluated in the context of pediatric brain injury. Stromal Cell-Derived Factor 1, a mediator of MSC and UCBC mobilization, is upregulated following neonatal HI injury (71, 73). Moreover, intracranial injection of SDF1 decreased inflammation, promoted re-myelination, and improved spatial learning following neonatal HI (70). Similarly, SCF is upregulated in neurons after brain injury and induces the migration of proliferating NSCs to areas of injured tissue (72). Treatment with SCF following HI in 7-day-old rats resulted in decreased brain tissue atrophy and improved motor outcomes (111), suggesting that both SDF1 and SCF would likely be similarly beneficial for the treatment of pediatric TBI. Thus, future research should place more emphasis on developing strategies to target the survival and maturation of endogenous stem cells for the treatment of pediatric TBI.

## Author Contributions

All authors listed have made a substantial, direct and intellectual contribution to the work, and approved it for publication.

## Conflict of Interest

The authors declare that the research was conducted in the absence of any commercial or financial relationships that could be construed as a potential conflict of interest.
